# The Relationship Between Improvement in Insomnia Severity and Long-Term Outcomes in the Treatment of Chronic Fatigue

**DOI:** 10.3389/fpsyg.2018.01764

**Published:** 2018-09-21

**Authors:** Daniel Vethe, Håvard Kallestad, Henrik B. Jacobsen, Nils Inge Landrø, Petter C. Borchgrevink, Tore C. Stiles

**Affiliations:** ^1^Department of Mental Health, Norwegian University of Science and Technology, Trondheim, Norway; ^2^Division of Mental Health Care, St. Olav’s University Hospital, Trondheim, Norway; ^3^Hysnes Rehabilitation Center, St. Olav’s University Hospital, Trondheim, Norway; ^4^National Competence Center for Pain and Complex Disorders, St. Olav’s University Hospital, Trondheim, Norway; ^5^Department of Pain Management and Research, Oslo University Hospital, Oslo, Norway; ^6^Department of Circulation and Medical Imaging, Norwegian University of Science and Technology, Trondheim, Norway; ^7^Department of Psychology, University of Oslo, Oslo, Norway; ^8^Department of Psychology, Norwegian University of Science and Technology, Trondheim, Norway

**Keywords:** chronic fatigue, insomnia, sleep, treatment, psychiatric symptoms

## Abstract

**Background:** The current treatments of choice for patients with chronic fatigue are moderately effective. One way to advance treatments is identifying process variables associated with good treatment outcomes. There is little knowledge regarding a possible association between insomnia and long-term outcomes in the treatment of chronic fatigue.

**Aims:** Testing whether (1) improvement in insomnia is associated with improvement in levels of fatigue at 1-year follow-up, and (2) if such a relationship remains when controlling for improvement in levels of anxiety and depression, and pain in patients with chronic fatigue.

**Methods:** Patients having been on sick leave 8 weeks or more due to chronic fatigue were referred to a return-to-work program. They received an intensive 3.5-week inpatient treatment program based on acceptance and commitment therapy (ACT). Before treatment and at 1-year follow-up the patients completed questionnaires assessing levels of insomnia severity, pain, anxiety and depression, and fatigue.

**Results:** A regression analysis found that changes in insomnia-severity were associated with changes in fatigue-levels at 1-year follow-up. When changes in levels of anxiety and depression were entered in the regression analysis, anxiety and depression was significantly associated with levels of fatigue but insomnia was not. The association between anxiety and depression and fatigue was at a trend level when pain was entered into the model.

**Conclusion:** Long-term improvement in insomnia severity was significantly associated with long-term improvement in chronic fatigue, but not independently of long-term improvement in anxiety and depression, and pain.

**Trial Registration:** https://clinicaltrials.gov/, identifier NCT01568970.

## Introduction

Chronic fatigue syndrome (CFS) is characterized by severe fatigue of a persistent or relapsing nature, prevailing for 6 months or more with a defined onset (Fukuda et al., 1994). The condition cannot be explained by any other medical problems, is not alleviated by rest, and leads to serious impairment in the domains of occupation, education, social or personal activity. A recent meta-analysis estimated a pooled prevalence of 3.28% in studies using self-report measures, and 0.76% in studies using clinical assessment (Johnston et al., 2013).

Research has largely focused on two forms of treatment demonstrating effect among patients with CFS: cognitive behavioral therapy (CBT) and graded exercise therapy (GET). CBT has been adapted and tested with CFS patients yielding superior results compared to standard medical treatment, relaxation, and adaptive pacing therapy (Sharpe et al., 1996; Deale et al., 1997; White et al., 2011), with moderate effect sizes (*d* = 0.48) (Malouff et al., 2008). GET has yielded effect sizes similar to that of CBT (*g* = 0.61) (Marques et al., 2015). Given the moderate effect sizes of CBT and GET, further exploration of factors associated with improvement and response to treatment is warranted.

A core problem in CFS, which may be relevant as a predictor of treatment outcome, is insomnia. Insomnia is defined as subjective problems with initiating or maintaining sleep, or non-restorative sleep, which leads to impaired daytime functioning (American Psychiatric Association, 2013). Daytime symptoms of insomnia include fatigue, low energy, cognitive impairments, and headache. For patients with CFS, non-restorative sleep is the most common insomnia-symptom with a prevalence ranging from 87.5 to 95.4% (Jason et al., 1999; Nisenbaum et al., 2003; Hamaguchi et al., 2011). Non-restorative sleep in CFS is associated with longer illness-duration and higher fatigue severity scores (Nisenbaum et al., 2003). Difficulty initiating sleep and maintaining sleep has been associated with both fatigue severity and greater global disability (Morriss et al., 1997). Moreover, subjective sleep-quality, but not actigraphy-defined sleep, has been found to predict next day fatigue levels (Russell et al., 2016). The subjective sleep problems in CFS overlap with the clinical features of insomnia. Therefore, insomnia may be highly prevalent in chronic fatigue, and the fatigue experienced by these patients might be partly explained by subjective insomnia symptoms.

To address the prevalent subjective sleep problems, sleep advice is routinely a component of CBT in randomized controlled trials in the CFS patient-group (Deale et al., 1997). Yet the number of treatment studies reporting sleep outcomes at end of treatment and follow-up is low. Only three treatment studies targeting chronic fatigue that we are aware of, have reported long-term changes in subjective sleep problems. In the first study, CBT and GET has been found to improve subjective sleep-disturbance significantly more than control-conditions at 1-year follow-up (White et al., 2011). In the same treatment study, sleep problems measured at 12 weeks were also found to mediate part of the effect of CBT and GET on fatigue at 1-year follow-up, when compared to standard medical care (Chalder et al., 2015). In the second study, a pragmatic rehabilitation treatment yielded improvements in subjective sleep-problems compared to standard medical treatment at 1-year follow-up (Powell et al., 2001). In the last study, a nurse-led home-based pragmatic rehabilitation was found to result in short-term, but not long-term, improvements in subjective sleep (Wearden et al., 2010). Thus, while there is focus on alleviating insomnia in treatments of chronic fatigue, there is unclear and limited knowledge about its impact on long-term treatment outcomes.

There is, however, evidence that pain, depression, and anxiety complicate the course for patients with CFS (Bentall et al., 2002; Cairns and Hotopf, 2005; Knoop et al., 2007; Kempke et al., 2010; Flo and Chalder, 2014). A recent study identified a CFS symptom-cluster characterized by anxiety, pain, and being focused on symptoms, to be associated with inferior treatment-outcome (Cella et al., 2011). Because insomnia is both its own diagnostic entity and a symptom of other conditions such as pain, depression, and anxiety, it is important to disentangle the relative contribution of these conditions and test if they have a unique impact on long-term treatment outcomes in chronic fatigue. Kallestad et al. (2015) found a strong association between improvement in insomnia severity and improvement in levels of fatigue from pre- to post-treatment in the same sample as the current study. At post-treatment improvement in insomnia severity had a unique association with improvement in fatigue, above and beyond the impact of improvements in levels of anxiety and depression and levels of pain. To our knowledge there have been no studies testing the association between improvement in insomnia severity and improvement in levels of fatigue long-term.

Thus, the aim of the current study is therefore to test the long-term relationship between improvement in insomnia-symptoms and levels of fatigue for patients with chronic fatigue who have received treatment. Our hypotheses were (1) long-term improvement in insomnia-symptoms are significantly associated with improvement in fatigue at 1-year follow-up, and (2) such a relationship remains when controlling for improvement in levels of anxiety and depression, and levels of pain.

## Materials and Methods

### Setting

A repeated measures treatment-study was conducted on a sample of patients with fatigue, who had been referred to a return to work (RTW) program by their general practitioner. Patients were consecutively recruited from January 2012 to June 2013, with an outpatient multidisciplinary team consisting of a psychologist, a physician, and a physiotherapist evaluating which patients met the criteria for inclusion in the program, and hence also in the study. Prior to evaluation at the outpatient clinic, the patients had been asked to complete 18 different questionnaires (386 items), using an online self-report survey. At the termination of treatment, and at 1-year follow-up, the participants were again asked to complete six of these online-questionnaires.

### Participants

The inclusion criteria for patients in the RTW-program were age between 18 and 60 years and to have been on sick leave at least 8 weeks due to musculoskeletal disorders, pain, fatigue, and/or common mental disorders. Moreover, a desire to increase participation in the workforce was required, along with having been adequately assessed and treated for any physical health problems prior to participation, and to be able to attend rehabilitation between 08:30 a.m. and 03:00 p.m. on weekdays.

Exclusion criteria were substance abuse and addiction, severe mental illness (ongoing mania, psychosis, or suicidal ideation), pregnancy, and unexpressed difficulty functioning in a group. Further, patients in need of 24-h personal assistance or unable to communicate in Norwegian were not accepted for rehabilitation.

Extending on the criteria for the RTW-program, the participants included in the current study had to report fatigue lasting more than 6 months, and score equal to or more than 5 on the Chalder Fatigue Scale (Chalder et al., 1993), thus being considered to have chronic fatigue. Furthermore, to be included in all the steps of analysis, the patients had to be exempt from any missing data on the variables in the hierarchic regression model. All patients were ultimately part of a larger clinical trial (Fimland et al., 2014).

### Treatment

The patients were offered a 3.5-week comprehensive, multidisciplinary, treatment-program at Hysnes rehabilitation center, a branch of St. Olav’s Hospital in Trondheim, Norway. The treatment program was based on acceptance and commitment therapy (ACT) principles, with the details of this program already described elsewhere (Fimland et al., 2014). The treatment was structured in 7-h days and was given over a period of 17 weekdays. Both individual and group-based treatment sessions were utilized to facilitate improvement, with group-sessions being most frequent. Group sessions spanned topics of socialization to the ACT-model and motivation for change, barriers and the issue of control, consequences of trying to control symptoms, family and important supporters, cognitive defusion (you are not your thoughts), communication and conflict, language, and staying committed to value-guided behavior. The individual sessions emphasized the identification of goals and values, and facilitating the commitment to these.

The multidisciplinary team of therapists had extensive and differing backgrounds in physical therapy, psychology, exercise physiology, medicine, and nursing. All the therapists were trained and supervised in the ACT-model, and were titled RTW coordinators. Each coordinator was responsible for assisting two or three participants through the program, and targeted three main areas in the individual sessions: mental training, physical training, and work-related problem solving. Further, three multidisciplinary team-meetings were arranged coinciding with the inpatient stay, giving the coordinators a platform to discuss strategies for handling participants’ obstacles and possibilities with regards to returning to work.

### Assessments

#### Psychological and Medical Examination

A licensed clinical psychologist assessed comorbid mental disorders using the Structured Clinical Interview for DSM-IV (SCID-IV) (First et al., 1995). A physician assessed the patients’ current medication use and reviewed their medical records.

#### Fatigue

The presence and severity of fatigue was assessed using the Chalder Fatigue Scale (Chalder et al., 1993), an 11-item questionnaire gauging both physical and mental fatigue. Each item comprised of four response categories scored bimodally 0-0-1-1 (e.g., 0 = better than usual; 0 = no more than usual; 1 = worse than usual; 1 = much worse than usual). A score of 5 or higher indicates chronic fatigue. The Chalder Fatigue Scale has yielded high reliability and validity scores (Chalder et al., 1993).

#### Pain

Pain level was assessed using one item from the Short Form-8 (SF-8) describing average pain the last 7 days. This was indicated on a Likert scale from 1 = no pain, to 6 = very strong pain. This item has been found valid as a self-report measure of pain in a large Norwegian cohort (Landmark et al., 2012).

#### Depression and Anxiety

To assess levels of anxiety and depression the Hospital Anxiety and Depression Scale (HADS) was employed. The HADS consists of 14 self-report items, where seven items are intended to assess symptoms of depression and seven items to assess symptoms of anxiety. The validity of the HADS for use with CFS-patients has been documented in at least one study (McCue et al., 2006).

#### Insomnia

The Insomnia Severity Index (ISI) was applied to register the levels of insomnia symptoms. Seven items measuring the nature, severity and impact of insomnia symptoms make up the ISI. [The items are as follows: (1) difficulty falling asleep, (2) difficulty maintaining sleep, (3) early morning awakenings, (4) satisfaction/dissatisfaction with sleep pattern, (5) interference of sleep problems with daily functioning, (6) sleep problems being noticeable by others, and (7) levels of distress/worry caused by the sleep problems]. A five-point Likert scale is used to indicate level of severity on each item (e.g., 0 = no problem; 4 = very severe problem), giving a maximum total score of 28. Reliability and validity has been established for the ISI (Bastien et al., 2001; Morin et al., 2011), and it is a recommended outcome measure for insomnia severity in clinical trials (Buysse et al., 2006).

### Ethics Statement

All subjects gave written informed consent in accordance with the Declaration of Helsinki. The protocol was approved by the Regional Ethical Committee for Research in Health in Trondheim, Norway (ref. no.: 2012/1241).

### Statistics

A cut-off score of ISI > 14 was used to determine clinically significant insomnia-cases. A cut-off of ISI < 8 was used to identify normal sleepers.

All included variables had values of asymmetry and kurtosis between -2 and +2, and could therefore be considered normally distributed (George and Mallery, 2010). Paired samples *t*-tests were performed to measure differences in variables from before treatment to 1-year follow-up.

A hierarchical regression model was applied to test both hypotheses. We entered the following variables in 9 steps: (1) age, (2) gender, (3) pre-treatment levels of fatigue, (4) pre-treatment levels of insomnia, (5) pre-treatment levels of anxiety and depression, (6) pre-treatment levels of pain, (7) follow-up levels of insomnia, (8) follow-up levels of anxiety and depression, and (9) follow-up levels of pain. The dependent variable was level of fatigue at 1-year follow-up. This sequence of variables in the regression model tests if changes in insomnia severity, levels of anxiety and depression, and/or pain-levels, is independently associated with changes in levels of fatigue from pre-treatment to follow-up, controlling for age and gender.

## Results

### Descriptive Data

During the inclusion period 279 patients were offered treatment. Chronic fatigue was reported by a subset of 188 of these patients, and 104 patients completed assessments both before treatment and at 1-year follow-up. Furthermore, 15 of these patients were identified as having comorbid mental disorders before treatment using the SCID-interview, and were excluded from further analyses to obtain a pure sample of patients with chronic fatigue. A total of 89 patients thus constituted the final sample for analyses. The patients were between 22 and 61 years of age (*SD* = 9.0). There were 76 females (85.4%) and 13 males (14.6%).

Pre-treatment, 32 (36.0%) patients had clinically significant insomnia, whereas on 1-year follow-up, 17 (19.1%) patients had clinically significant insomnia. Similarly at pre-treatment, 19 (21.3%) patients were normal sleepers, and at follow-up, 39 (43.8%) patients were normal sleepers.

See **Table 1** for pre-treatment and 1-year follow-up means in fatigue, anxiety and depression, pain, and insomnia-scores.

**Table 1:** Changes in clinical variables for 89 patients from before receiving 3-weeks of group-based acceptance therapy to 1 year after treatment termination.

Variables	Pre-treatment		1-year follow-up		Paired samples *t*-test		
	*M*	*SD*	*M*	*SD*	*t*	*p*	*d*
Fatigue	9.07	1.83	5.67	4.00	8.1	<0.001	1.09
Insomnia severity	12.25	5.92	9.03	5.92	5.8	<0.001	0.54
Pain	3.99	1.10	3.71	1.27	2.0	>0.05	0.24
Anxiety and depression	14.97	6.85	10.38	6.56	6.2	<0.001	0.68


### Hypothesis Testing

A summary of the hierarchical regression analysis testing associations with levels of fatigue at follow-up is displayed in **Table 2**. The regression model explained 29.3% of the variance in levels of fatigue at 1-year follow-up.

**Table 2:** Summary of the hierarchical regression analysis testing predictors of changes in fatigue levels for 89 patients with chronic fatigue.

Step	Variables	Δ*R*^2^	*B*	SE	β	*t*	
1	Age	0.011	0.046	0.047	0.105	0.986	
2	Age	0.010	0.044	0.047	0.100	0.936	
	Gender	0.010	-1.136	1.189	-0.102	-0.956	
3	Age	0.010	0.044	0.046	0.099	0.947	
	Gender	0.009	-1.041	1.163	-0.094	-0.895	
	Fatigue pre-treatment	0.054	0.502	0.226	0.232	2.221	^∗^
4	Age	0.013	0.051	0.046	0.116	1.109	
	Gender	0.010	-1.093	1.160	-0.098	-0.942	
	Fatigue pre-treatment	0.062	0.548	0.228	0.253	2.400	^∗^
	Insomnia pre-treatment	0.017	-0.089	0.071	-0.133	-1.252	
5	Age	0.014	0.052	0.046	0.119	1.130	
	Gender	0.010	-1.126	1.165	-0.101	-0.966	
	Fatigue pre-treatment	0.067	0.595	0.240	0.275	2.477	^∗^
	Insomnia pre-treatment	0.009	-0.070	0.077	-0.104	-0.903	
	Anxiety and depression pre-treatment	0.005	-0.045	0.070	-0.079	-0.651	
6	Age	0.017	0.058	0.046	0.132	1.264	
	Gender	0.021	-1.686	1.189	-0.151	-1.418	
	Fatigue pre-treatment	0.039	0.472	0.246	0.218	1.920	^+^
	Insomnia pre-treatment	0.014	-0.088	0.077	-0.133	-1.154	
	Anxiety and depression pre-treatment	0.005	-0.048	0.069	-0.083	-0.699	
	Pain pre-treatment	0.036	0.742	0.404	0.207	1.837	^+^
7	Age	0.010	0.044	0.044	0.099	1.004	
	Gender	0.012	-1.262	1.130	-0.113	-1.117	
	Fatigue pre-treatment	0.032	0.427	0.233	0.197	1.834	^+^
	Insomnia pre-treatment	0.078	-0.252	0.088	-0.378	-2.873	^∗∗^
	Anxiety and depression pre-treatment	0.002	-0.027	0.065	-0.048	-0.421	
	Pain pre-treatment	0.012	0.444	0.392	0.124	1.133	
	Insomnia follow-up	0.103	0.280	0.085	0.420	3.299	^∗∗^
8	Age	0.009	0.042	0.042	0.095	0.982	
	Gender	0.011	-1.226	1.102	-0.110	-1.113	
	Fatigue pre-treatment	0.041	0.487	0.228	0.225	2.132	^∗^
	Insomnia pre-treatment	0.041	-0.191	0.090	-0.287	-2.135	^∗^
	Anxiety and depression pre-treatment	0.020	-0.108	0.073	-0.187	-1.490	
	Pain pre-treatment	0.008	0.369	0.383	0.103	0.962	
	Insomnia follow-up	0.024	0.160	0.098	0.239	1.630	
	Anxiety and depression follow-up	0.048	0.179	0.078	0.297	2.302	^∗^
9	Age	0.005	0.033	0.043	0.076	0.773	
	Gender	0.008	-1.023	1.118	-0.092	-0.916	
	Fatigue pre-treatment	0.047	0.528	0.232	0.244	2.281	^∗^
	Insomnia pre-treatment	0.035	-0.178	0.090	-0.267	-1.970	^+^
	Anxiety and depression pre-treatment	0.016	-0.097	0.073	-0.167	-1.317	
	Pain pre-treatment	0.002	0.195	0.417	0.054	0.467	
	Insomnia follow-up	0.019	0.144	0.099	0.216	1.455	
	Anxiety and depression follow-up	0.035	0.159	0.080	0.263	1.979	^+^
	Pain follow-up	0.010	0.382	0.364	0.123	1.049	


*The regression model explains 29.3% of variance in fatigue at 1-year follow-up. Dependent variable: sum score on the Chalder Fatigue Scale 1-year follow-up. Fatigue = sum score on the Chalder Fatigue Scale. Pain = score on level of somatic pain from the Short-Form Health Status-8. Depression and anxiety = sum score on the Hospital Anxiety and Depression Scale. Insomnia = sum score on the Insomnia Severity Index. ^+^p < 0.10, ^∗^p < 0.05, ^∗∗^p < 0.01.*

Long-term changes in levels of insomnia severity were significantly associated with improvement in levels of fatigue from pre-treatment to follow-up (step 7). When controlling for changes in levels of anxiety and depression (step 8), this associating was no longer significant. Anxiety and depression was, however, associated with the outcome. Changes in levels of pain were not associated with improvement in levels of fatigue (step 9), but anxiety and depression remained associated with the outcome at a trend level in the fully adjusted model. Only pre-treatment levels of fatigue were significantly associated with follow-up levels of fatigue in the last step of the analysis.

The hierarchical regression model yielded significant regression equations in steps 7, 8, and 9. The introduction of follow-up insomnia-severity in step 7 resulted in a significant increase in *R*^2^, and the model explained 23.5% (adjusted *R*^2^= 0.17) of the variation in fatigue-level change [*F*(7,81) = 3.56, *p* < 0.01]. In step eight, the addition of follow-up levels of anxiety and depression significantly increased the *R^2^*, and the model explained 28.3% (adjusted *R^2^* = 0.21) of the variation in fatigue-level change [*F*(8,80) = 3.94, *p* < 0.001]. The introduction of follow-up levels of pain in the last step did not significantly increase the *R^2^*, and the model now explained a total of 29.3% (adjusted *R^2^* = 0.21) of the variation in fatigue-level change [*F*(9,79) = 3.63, *p* < 0.001].

Due to a large number of patients not completing the follow-up assessments, *t*-tests were performed to uncover any pre-treatment or post-treatment differences between the participants who completed assessments at follow-up compared to participants who completed assessments post-treatment, but not at follow-up. Results are summarized in **Supplementary Table 1**. The missing at random assumption is not supported for participant data at follow-up, as a consequence of significant differences between completers and non-completers in levels of insomnia and fatigue post-treatment.

## Discussion

We found that patients with chronic fatigue who received a 3-week intensive ACT-based treatment, experienced large improvements in fatigue levels from pre-treatment assessment to follow-up assessment 1 year after treatment termination. Our hypotheses for this study was (1) that long-term improvement in insomnia severity would be associated with long-term improvement in levels of fatigue, and (2) that this association would be independent of long-term changes in levels of anxiety and depression and pain. We found support for the first hypothesis but not the second. Changes in insomnia severity were associated with changes in levels of fatigue at follow-up. This result was, however, not significant when controlling for long-term changes in levels of anxiety and depression. At the same time, long-term changes in anxiety and depression were independently associated with changes in levels of fatigue when controlling for long-term changes in insomnia severity. This association was only at a trend level when also controlling for long-term changes in levels of pain.

The current finding extends on the results published by Kallestad et al. (2015) regarding change from pre-treatment to post-treatment in the same sample as the current study. In their study, changes in both insomnia severity, and levels of anxiety and depression during treatment, were independently associated with fatigue-levels immediately after treatment. At 1-year follow-up the association between long-term changes in insomnia severity and levels of fatigue disappears when controlling for long-term changes in levels of anxiety and depression, suggesting that participants with improvement in insomnia severity often experienced an improvement in anxiety and depression. Further, it seems that improvement in anxiety and depression explains additional variance over and beyond the shared contributions of the two variables. This may suggest that targeting the symptoms of anxiety and depression in treatment may be more relevant than insomnia for long-term improvement in fatigue. It does, however, not exclude the possibility that by adding evidence-based specific interventions to improve sleep, one can increase the unique associations between changes in insomnia severity and changes in fatigue levels from pre-treatment to post-treatment and furthermore also produce unique associations during the follow-up period because then the patients can be capable of continuing to apply specific strategies to improve sleep themselves and not only relying on more general strategies to control symptoms. The latter is only a hypothesis that has to be tested empirically.

While insomnia is highly prevalent in patients with chronic fatigue, few have studied its role in treatment. Only three studies we are aware of, have investigated the long-term outcomes of sleep-problems in treatment of chronic fatigue. Two RCTs have reported outcomes for sleep-problems after pragmatic rehabilitation interventions targeting CFS (Powell et al., 2001; Wearden et al., 2010). The first RCT compared pragmatic rehabilitation to standard medical care. The intervention led to improved physical function and fatigue, and significant improvement in subjective sleep problems at 1-year follow-up (Powell et al., 2001). The authors did not report outcomes for sleep problems at their 2-year follow-up, but treatment gains seemed maintained on physical functioning and fatigue measures (Bentall et al., 2002). The second RCT, compared a nurse-led pragmatic rehabilitation intervention to supportive listening, and general practitioner treatment as usual (Wearden et al., 2010). A significant improvement in sleep was found for pragmatic rehabilitation post-treatment, but not at 1-year follow-up, compared to treatment as usual. At 1-year follow up, the pragmatic rehabilitation had no effect on any of the outcome measures. These studies suggest pragmatic rehabilitation has a positive effect on sleep-problems short-term, but the long-term effect seem unclear. Moreover, these studies did not report analyses regarding the relationship between improvement in sleep and improvement primary outcomes. A recent systematic review focusing on the effect of CBT and GET on sleep in patients with CFS (Russell et al., 2017), points to a need to further understand whether improved sleep may be one mechanism by which treatments influence symptoms and daytime functioning.

Another RCT reporting outcomes for sleep-problems is the PACE-trial (White et al., 2011). Though recently questioned on its scientific rigor (Wise, 2016), it is still the largest treatment study on patients with CFS. Regarding sleep, the authors reported a reduction in sleep-problems from baseline to follow-up (White et al., 2011). The improvement in sleep corresponded in size to that reported by patients in our study. Further, the secondary analysis of the PACE-trial data indicated that levels of sleep problems at 12-weeks (mid-treatment) mediated a portion of the long-term (1-year) treatment effect for CBT and GET vs. standard medical care (Chalder et al., 2015). It is worth noting that depression also mediated a proportion of the treatment effect for CBT vs. standard medical care. These mediating effects were small, however, compared to those of other variables (e.g., fear-avoidance beliefs). The secondary analysis of the PACE-trial tested potential mediators in separate models, not taking into account the likely overlap of these variables. In our study we identify a covariance in the improvement of symptoms, and therefore find that in explaining fatigue-outcome, the improvement in sleep is closely related to that of improvement in anxiety and depression.

Interestingly, we found in our study that long-term changes in levels of anxiety and depression are independently associated with long-term changes in levels of fatigue when controlling for long-term changes in insomnia severity. When controlling for changes in levels of pain, the association with fatigue was at a trend level. Previous research has also found anxiety and depression as measured by the HADS to be related to poor treatment outcome (Bentall et al., 2002), and more functional impairment at follow-up (Sharpe et al., 1992). Depression, as assessed by another self-report measure, has also been associated with worse disability in a range of domains, and was found to completely mediate the relation between fatigue and psychosocial disability in particular (Hadlandsmyth and Vowles, 2009). The current study excluded participants diagnosed with anxiety or depression after SCID-interviews, suggesting that even subclinical levels of anxiety and depression may influence fatigue. Our finding is in line with the results from previous publications, indicating that improvement in anxiety and depression is important for good outcome in the treatment of chronic fatigue.

A role for pain in explaining response to treatment for CFS has been identified in previous research, with patients experiencing more pain before treatment benefitting less from treatment (Knoop et al., 2007; Cella et al., 2011). However, we found little support for a role of pain in the long-term improvement of fatigue. Neither pre-treatment levels of pain, nor improvement in pain, was associated with improvement in levels of fatigue. It could be that the non-significant improvement in pain-severity in our study was not enough to influence levels of fatigue.

In sum, our findings extend on previous research by focusing on long-term outcomes in fatigue, and how it relates to changes in pain, anxiety and depression, and insomnia. Our study suggests that long-term, improvement in insomnia may be related to improvement in fatigue, but that this association is explained by long-term improvements in levels of anxiety and depression. Most importantly, previous studies have relied on separate analyses of how changes in sleep and depression and anxiety, impact treatment outcomes. This may have led to an exaggerated estimation of the impact of these variables, as there is an overlap in the variance they explain in long-term improvements in fatigue.

### Limitations

There are several limitations to this study. First, clinical assessment is the gold standard for evaluating psychiatric symptoms. In the current study, only self-report measures have been used. However, the questionnaires included have been widely used in research to identify symptoms and track changes in severity during treatment (Buysse et al., 2006; McCue et al., 2006; Landmark et al., 2012).

Second, this study did not use a clinical diagnostic assessment to identify CFS cases. Instead, self-report on the Chalder Fatigue Scale was used as a marker for chronic fatigue, in addition to describing the fatigue as lasting more than 6 months. A recent study found patients referred for chronic unexplained fatigue only received a CFS diagnosis in 23.3% of the cases after a multidisciplinary diagnostic assessment (Mariman et al., 2013), suggesting the findings in our study may not be generalizable to patients meeting the full criteria for a CFS diagnosis.

Third, the nature of the repeated measures design used in the current study cannot discriminate between improvement on variables due to treatment effect, and improvement caused by confounding variables or regression to the mean. However, in a clinical population characterized by chronic symptoms, the large effect size on the main outcome of fatigue indicates a real treatment effect.

Fourth, it is not known whether the patients received any other treatment or medication between post-treatment and follow-up. It can therefore not be excluded that some patients have had improvement due to concurrent treatment.

Fifth, the rate of patients missing complete datasets at follow-up was high in our sample. Eighty-nine out of 159 patients offered treatment (55%) had complete datasets on follow up. Loss of data of more than 20% can have a profound impact on the validity of a study, if this loss is not of a random nature (Kristman et al., 2004). Additional analyses found some differences between participants completing the follow-up questionnaires, and those who did not. Participants completing follow-up had significantly lower scores on fatigue levels and insomnia severity at post-treatment, compared to non-completers (**Supplementary Table 1**). Further, follow-up non-completers did not have a significant improvement in levels of insomnia or pain severity from pre-treatment to post-treatment. This non-random nature of missing participants at follow-up could lead to an overestimation of the effects in the current study, and thus constitutes a major limitation.

Sixth, due to the number of dropouts, the number of participants available for analysis on 1-year follow-up borders the acceptable limit for number of variables included in a multiple regression model (10 patients for each step in the regression model). The statistical power of the analysis is thus limited. With a higher number of participants at follow-up, statistical significance might have been reached on variables identified as trends in the current study. The presence of false negatives can therefore not be ruled out in this study.

Seventh, a self-report measure was used to assess sleep in the current study. As previous research has often failed to find objectively poor sleep using PSG (Watson et al., 2003; Majer et al., 2007), the current study focused on subjectively poor sleep. However, some recent studies have identified PSG-defined poor sleep in CFS-patients compared to healthy controls (Gotts et al., 2016; Tobback et al., 2016). Therefore, subjective sleep-measures may need to be supplemented by objective sleep-measures in the future.

Finally, since the study primarily made use of a specific treatment modality (ACT), it is possible that the results are specific to the interventions used in this study. Thus, replication is called for using another treatment approach.

## Conclusion

Long-term improvement in insomnia severity was significantly associated with long-term improvement in chronic fatigue, but not independently of long-term improvement in anxiety and depression, and pain.

## Author Contributions

DV, TS, and HK identified the study hypotheses and performed the data analyses. DV, HK, TS, and HJ have contributed to writing the manuscript. The treatment trial was designed by TS, NL, and PB. All authors have read, contributed to the interpretation of results, and critically revised the manuscript.

## Conflict of Interest Statement

The authors declare that the research was conducted in the absence of any commercial or financial relationships that could be construed as a potential conflict of interest.
