# Supplementary material for: The Relationship Between Improvement in Insomnia Severity and Long-Term Outcomes in the Treatment of Chronic Fatigue
Source: Front Psychol. 2018 Sep 21;9:1764. doi: 10.3389/fpsyg.2018.01764 (PMC6160743; doi:10.3389/fpsyg.2018.01764)
Supplement: Supplementary file 1 [file Table_1.DOCX]

Supplementary Material

**The relationship between improvement in insomnia severity and long-term outcomes in the treatment of Chronic Fatigue**

**Daniel Vethe^*^, Håvard Kallestad, Henrik B. Jacobsen, Nils Inge Landrø, Petter C. Borchgrevink, Tore C. Stiles**

*** Correspondence:** Corresponding Author: daniel.vethe@ntnu.no

**Supplement 1.** Summary of results from t-tests comparing participants not completing follow-up to participants completing follow-up on pre-treatment and post-treatment measures.

| Variable | Not completed follow-up | |  | Completed follow up | |  | Independent samples t-test | |
| --- | --- | --- | --- | --- | --- | --- | --- | --- |
|  | *M* | *SD* |  | *M* | *SD* |  | *t* | *p* |
| Fatigue pre-treatment | 9.43 | 1.29 |  | 9.01 | 1.83 |  | 1.0 | n.s. |
| Insomnia pre-treatment  Anxiety and depression pre-treatment  Pain pre-treatment  Fatigue post-treatment  Insomnia post-treatment  Anxiety and depression post-treatment | 14.00  16.54  3.93  7.96  13.18  12.46 | 6.82  8.24  1.12  2.53  7.72  8.10 |  | 12.25  14.97  3.99  5.84  9.13  11.05 | 5.93  6.85  1.10  4.01  5.36  6.10 |  | 1.3  1.0  -0.3  3.2  2.6  1.0 | n.s.  n.s.  n.s.  <.01  <.05  n.s. |
| Pain post-treatment | 3.68 | 1.25 |  | 3.56 | 1.27 |  | 0.5 | n.s. |
